# *Trypanosoma livingstonei*: a new species from African bats supports the bat seeding hypothesis for the *Trypanosoma cruzi* clade

**DOI:** 10.1186/1756-3305-6-221

**Published:** 2013-08-03

**Authors:** Luciana Lima, Oneida Espinosa-Álvarez, Patrick B Hamilton, Luis Neves, Carmen SA Takata, Marta Campaner, Márcia Attias, Wanderley de Souza, Erney P Camargo, Marta MG Teixeira

**Affiliations:** 1 Departamento de Parasitologia, Instituto de Ciências Biomédicas, Universidade de São Paulo, São Paulo, SP 05508-900, Brazil; 2 Biosciences, College of Life and Environmental Sciences, University of Exeter, Exeter, UK; 3 Centro de Biotecnologia, Universidade Eduardo Mondlane, Maputo, Mozambique; 4 Laboratório de Ultraestrutura Celular Hertha Meyer, Instituto de Biofísica Carlos Chagas Filho, Universidade Federal do Rio de Janeiro, Rio de Janeiro, RJ 21941-902, Brazil

**Keywords:** Chiroptera, Taxonomy, Phylogeny, Phylogeography, Evolution, Africa, *Trypanosoma cruzi*

## Abstract

**Background:**

Bat trypanosomes have been implicated in the evolutionary history of the *T. cruzi* clade, which comprises species from a wide geographic and host range in South America, Africa and Europe, including bat-restricted species and the generalist agents of human American trypanosomosis *T. cruzi* and *T. rangeli*.

**Methods:**

Trypanosomes from bats (*Rhinolophus landeri* and *Hipposideros caffer*) captured in Mozambique, southeast Africa, were isolated by hemoculture. Barcoding was carried out through the V7V8 region of Small Subunit (SSU) rRNA and Fluorescent Fragment Length barcoding (FFLB). Phylogenetic inferences were based on SSU rRNA, glyceraldehyde phosphate dehydrogenase (gGAPDH) and Spliced Leader (SL) genes. Morphological characterization included light, scanning and transmission electron microscopy.

**Results:**

New trypanosomes from bats clustered together forming a clade basal to a larger assemblage called the *T. cruzi* clade. Barcoding, phylogenetic analyses and genetic distances based on SSU rRNA and gGAPDH supported these trypanosomes as a new species, which we named *Trypanosoma livingstonei* n. sp. The large and highly polymorphic SL gene repeats of this species showed a copy of the 5S ribosomal RNA into the intergenic region. Unique morphological (large and broad blood trypomastigotes compatible to species of the subgenus *Megatrypanum* and cultures showing highly pleomorphic epimastigotes and long and slender trypomastigotes) and ultrastructural (cytostome and reservosomes) features and growth behaviour (when co-cultivated with HeLa cells at 37°C differentiated into trypomastigotes resembling the blood forms and do not invaded the cells) complemented the description of this species.

**Conclusion:**

Phylogenetic inferences supported the hypothesis that *Trypanosoma livingstonei* n. sp. diverged from a common ancestral bat trypanosome that evolved exclusively in Chiroptera or switched at independent opportunities to mammals of several orders forming the clade *T. cruzi*, hence, providing further support for the bat seeding hypothesis to explain the origin of *T. cruzi* and *T. rangeli.*

## Background

Trypanosomes (Euglenozoa: Kinetoplastea: Trypanosomatidae) are blood parasites widespread in all continents, adapted to all classes of vertebrates and transmitted by leeches and a variety of bloodsucking arthropods. Although Chiroptera harbour numerous trypanosome species with a high prevalence and worldwide distribution, species diversity, vectors, life cycles, distribution and trypanosome evolution remain poorly understood [1-10].

The majority of trypanosomes reported in bats have not been cultivated, and their classification has been based exclusively on the morphology of blood trypomastigotes. Large blood trypanosomes of the subgenus *Megatrypanum,* followed by small blood forms of the subgenus *Schizotrypanum,* comprise the majority of the trypanosomes reported in bats throughout South America, Asia, Europe and, especially, Africa [1,3,5,7,8,10-14]. The subgenus *Megatrypanum,* originally comprising large blood trypanosomes from artiodactyls [15], was amended exclusively on a morphological basis to include any large trypanosome found in bats, monkeys and rodents [1,2,4,6]. Molecular phylogenetic analysis has demonstrated the polyphyly of the traditional subgenus *Megatrypanum*, which was revised as a clade comprising trypanosomes from ruminants headed by the type species *T. theileri,* a cosmopolitan parasite of cattle [16-19]. However, in the reappraisal of this subgenus, other species from non-ruminant hosts that putatively belong to this subgenus need to be phylogenetically positioned, especially those from bats, which together with trypanosomes from artiodactyls, account for most of the species assigned to this subgenus [1,4,7].

Most bat trypanosome species that have been characterised by molecular approaches belong to the subgenus *Schizotrypanum*[8-11,13,14,20-25]. With the exception of *T. cruzi,* there are no *Schizotrypanum* species in hosts other than bats. *T. rangeli* was found in Brazilian bats [26], this species is infective to several mammals and comprises distinct genotypes [26,27], which clustered into a clade containing *T. conorhini* from rats*, T. vespertilionis* from a European bat and two African trypanosomes from monkey and civet. Although *T. rangeli, T. conorhini* and *T. vespertilionis* were morphologically classified into the subgenera *Herpetosoma*, *Megatrypanum* and *Schizotrypanum*, respectively, molecular phylogenies demonstrated that they clustered together forming the strongly supported sister clade of the *Schizotrypanum* clade. *Trypanosoma* sp. (*T.* sp. bat) from an African megabat (suborder Megachiroptera) originally assigned to the subgenus *Megatrypanum* was positioned at the edge of this clade [8-10,18,20,23,24].

The major assemblage formed by the subgenus *Schizotrypanum* and the clade *T. rangeli/T. conorhini* was designated as the *T. cruzi* clade. The positioning of a kangaroo trypanosome at its edge in association with vicariance has supported the southern supercontinent hypothesis for the origin of *T. cruzi*. Accordingly, this species could have originated in marsupials at a time when South America, Australia and Antarctica formed a single continent. However, in conflict with this hypothesis, some Australian trypanosomes from marsupials are more related to trypanosomes from non-Australian hosts [9,18,20,21,23,24]. The discovery of African terrestrial mammals infected with trypanosomes placed in the *T. cruzi* clade [18] has complicated the southern supercontinent hypothesis.

Taken together, the findings that *T. c. marinkellei* from South American bats is the closest living relative of *T. cruzi*[8,10,22,25,28] followed by *T. erneyi* from African bats [10], the close phylogenetic relationship between *T. dionisii* from Europe and South America [8,9,24], the presence of *T. rangeli* in Brazilian bats [26] and the relationships of this species with African (*T*. sp. bat) and European (*T. vespertilionis*) bat trypanosomes, and the discovery of Tcbat, a bat-associated *T. cruzi* genotype found in South and Central America [14,22] all support the bat seeding hypothesis for the origin of the *T. cruzi* clade [24]. In this scenario, which is the most parsimonious for explaining the relationships observed within the *T. cruzi* clade, an ancestral trypanosome parasite in bats diverged to lineages that evolved exclusively in bats, giving rise to the bat-restricted species, or evolved through multiple switches at independent times in hosts of other mammalian orders, including the generalists *T. cruzi* and *T. rangeli*, which also infect bats. Multiple trypanosome jumps between hosts were most likely facilitated by the sharing of niches by bats, haematophagous insects (vectors) and terrestrial mammals. Oral infection through the predation of infected bats by other mammals and by the consumption of insect vectors by bats probably played important roles in the colonisation of new hosts by bat trypanosomes. Transmission of trypanosomes among bats is likely to occur by an oral route when the vector insects are eaten by insectivorous bats. The grooming habits of the bats probably facilitates the infection by the bat trypanosomes transmitted by ectoparasite cimicids [3,6,7]. Both vectorial and oral transmission routes are important in the natural transmission cycles of *T. cruzi* and other trypanosomes nested in the *T. cruzi* clade [1,3,6-8].

With the discovery of *T. erneyi*[10] and a new genotype of *T. dionisii* in the UK [9], bat trypanosomes from the Old World revealed to be more closely related to South American bat trypanosomes than showed by previous studies [8,22,26]. These findings suggested movement of bat trypanosomes between the New and Old worlds occurred in a relatively more recent time than bat fossil records suggested [9,24].

Trypanosomes from the *T. cruzi* clade are likely to have started to diversify sometime after the great diversification of bats in the Eocene (70–58 mya) [29-31]. However, the extant species of bat trypanosomes appear to have emerged during a short period and much more recently than expected based on the fragmented paleontological history of bats [9,24].

In this study, we isolated and characterised 14 new trypanosomes from African bats captured in Mozambique, southeast Africa, by inferring phylogenetic relationships using ribosomal SSU rRNA, gGAPDH and SL genes. Sequences from the new bat isolates were compared to those from other bat trypanosomes determined in this and in previous studies (including other isolates morphologically assignable to the subgenus *Megatrypanum*) to address taxonomic questions about bat trypanosomes. Comparison of bat trypanosomes by combining molecular, morphological and behavioural information provides new information on the evolutionary history of bat trypanosomes and the origin of the *T. cruzi* clade.

## Methods

### Collection sites, capture and identification of bats

**Figure 1:**
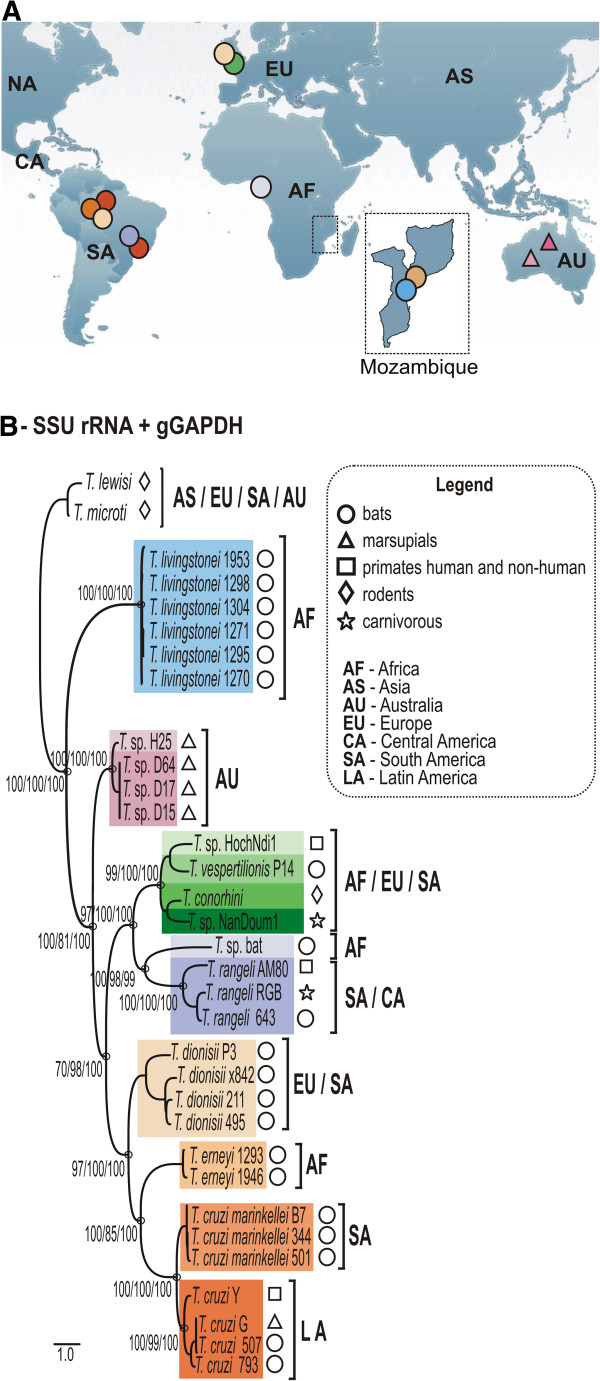
**Phylogeographical analysis of *T. livingstonei*, other bat trypanosomes and species from other mammals that nested into the clade *T. cruzi*. (a)** Geographic origin of all trypanosomes included in the phylogenetic analysis, with the map from Mozambique (south-eastern Africa) where *T. livingstonei* was isolated in detail. **(b)** ML phylogenetic analysis based on concatenated sequences of SSU rRNA and gGAPDH sequences (3.318 characters, –Ln = 12694.460743) from 6 *T. livingstonei* isolates*,* 20 isolates from other species of bat trypanosomes, and 13 trypanosomes from other mammalian orders; all the selected trypanosomes were previously positioned in the *T. cruzi* clade (GenBank accession numbers are listed on Table 1). Species from the *T. lewisi* clade were used as outgroups. Numbers are bootstrap values derived from 500 replicates in the P/ML/BI analyses.

Bats were captured in Mozambique, southeastern Africa, in the district of Chupanga (S18°02′ E35°34′), Zambezi valley, and the Gorongosa National Park (S18°58′ E34°21′), both of which are located in the Province of Sofala in central Mozambique (Table 1; Figure 1). Captures were carried out with mist nets; bats were anaesthetised and blood samples were collected by cardiac puncture as previously described [8,22]. For the molecular identification of bats, liver tissue samples were fixed in 100% ethanol, processed for genomic DNA and used to sequence the cytochrome b gene (Cyt b) as previously described [32]. Sequences were analysed by BLAST search in GenBank.

**Table 1:** ***Trypanosoma livingstonei *and other trypanosomes included in the combined gGAPDH and SSU rRNA analysis**

***Trypanosoma *TCC**^**a**^			**Host origin**	**Year**	**Geographic origin**	**GenBank acession number**		
						**SSU rRNA**	**gGAPDH**	**SL**
***T. livingstonei *isolates**								
1270	bat 12	bat	*Rhinolophus landeri*	2006	Mozambique (CH)	**KF192979**	**KF192958**	-
1271	bat 17	bat	*Rhinolophus landeri*	2006	Mozambique (CH)	**KF192980**	**KF192959**	-
1295	bat 29	bat	*Rhinolophus landeri*	2006	Mozambique (CH)	**KF192981**	**KF192960**	-
1298	bat 28	bat	*Rhinolophus landeri*	2006	Mozambique (CH)	**KF192982**	**KF192961**	-
1304	bat 20	bat	*Rhinolophus landeri*	2006	Mozambique (CH)	**KF192983**	**KF192962**	**KF192970/KF192971**
1902	CHMO 34	bat	*Rhinolophus landeri*	2009	Mozambique (CH)	**KF192985**^b^	**KF192963**	-
1933	CHMO 30	bat	*Rhinolophus landeri*	2009	Mozambique (CH)	**KF192986**^b^	**KF192964**	**KF192972/KF192973**
1935	MTR 16933	bat	*Rhinolophus landeri*	2009	Mozambique (CH)	**KF192987**^b^	**KF192965**	-
1947	CHMO 32	bat	*Rhinolophus landeri*	2009	Mozambique (CH)	**KF192988**^b^	**KF192966**	-
1948	CHMO 31	bat	*Rhinolophus landeri*	2009	Mozambique (CH)	**KF192989**^b^	**KF192967**	**KF192974/KF192975/KF192976**
1954	CHMO 33	bat	*Rhinolophus landeri*	2009	Mozambique (CH)	**KF192990**^b^	**KF192968**	-
1953	GOMO 28	bat	*Hipposideros caffer*	2009	Mozambique (GO)	**KF192984**	**KF192969**	-
2339	GOBAT 61	bat	*Hipposideros caffer*	2012	Mozambique (GO)	**KF192994**^b^	-	-
2348	GOBAT 58	bat	*Hipposideros caffer*	2012	Mozambique (GO)	**KF192993**^b^	-	-
**Other bat trypanosomes**								
	*T. vespertilionis* P14	bat	*Pipistrellus pipistrellus*	1972	England	AJ009166	AJ620283	AF116564
60	*T.* sp. bat (LV634)	bat	*Rousettus aegyptiacus*	1997	Gabon	AJ012418	GQ140365	**KF192977**
643	*T. rangeli*	bat	*Platyrrhinus lineatus*	2003	Brazil	FJ900242	GQ140364	EU867800
-	*T. dionisii* P3	bat	*Pipistrellus pipistrellus*	1971	England	AJ009151	AJ620271	AJ250744
-	*T. dionisii* x842	bat	*Nyctalus noctula*	2006	England	FN599058	FN599055	-
211	*T. dionisii*	bat	*Eptesicus brasiliensis*	2000	Brazil	FJ001666	GQ140362	-
495	*T. dionisii*	bat	*Carollia perspicillata*	2002	Brazil	FJ001667	GQ140363	-
1293	*T. erneyi*	bat	*Tadarida* sp.	2006	Mozambique (CH)	JN040987	JN040964	**KF192978**
1946	*T. erneyi*	bat	*Mopys condylurus*	2009	Mozambique (CH)	JN040989	JN040969	-
-	*T. c. marinkellei* B7	bat	*Phyllostomus discolor*	1974	Brazil	AJ009150	AJ620270	-
344	*T. c. marinkellei*	bat	*Carollia perspicillata*	2002	Brazil	FJ001664	GQ140360	-
501	*T. c. marinkellei*	bat	*Carollia perspicillata*	2002	Brazil	FJ001665	GQ140361	-
507	*T. cruzi* (TcI)	bat	*Carollia perspicillata*	2002	Brazil	FJ900240	GQ140352	-
793	*T. cruzi* (Tcbat)	bat	*Myotis levis*	2004	Brazil	FJ900241	GQ140358	-
**Trypanosomes of other mammals**								
-	*T*. sp. H25	kangaroo	*Macropus giganteus*	1997	Australia	AJ009168	AJ620276	-
-	*T*. sp. D15	possum	*Trichosurus vulpecula*	2009	Australia	JN315381	JN315395	-
-	*T*. sp. D17	possum	*Trichosurus vulpecula*	2009	Australia	JN315382	JN315396	-
-	*T*. sp. D64	possum	*Trichosurus vulpecula*	2009	Australia	JN315383	JN315397	-
-	*T*. sp. BRA2	bush rat	*Rattus fuscipes*	2007	Australia	FJ823117	-	-
-	*T.* sp. HochNdi1	monkey	*Cercopithecus nictitans*	2004	Cameroon	FM202493	FM164794	-
-	*T.* sp. NanDoum1	carnivore	*Nandinia binotata*	2004	Cameroon	FM202492	FM164793	-
25e	*T. conorhini*	rat	*Rattus rattus*	1947	Brazil	AJ012411	AJ620267	AJ272600
	*T. lewisi*	rat	*Rattus rattus*	1973	England	AJ009156	AJ620272	-
86	*T. rangeli* AM80	human	*Homo sapiens*	1996	Brazil	AY491766	JN040973	-
-	*T. rangeli* RGB	dog	*Canis familiaris*	1949	Venezuela	AJ009160	AF053742	AJ012419
34	*T. cruzi* Y (TcII)	human	*Homo sapiens*	1953	Brazil	AF301912	GQ140353	-
30	*T. cruzi* G (TcI)	marsupial	*Didelphis marsupialis*	1983	Brazil	AF239981	GQ140351	-
**Bat blood samples**								
-	bat 19	bat	*Rhinolophus landeri*	2006	Mozambique (CH)	**KF192992**^b^	-	-
-	bat 25	bat	*Rhinolophus landeri*	2006	Mozambique (CH)	**KF192991**^b^	-	-

^a^Number codes of cultures of trypanosomes cryopreserved in the Trypanosomatid Culture Collection (TCC).

Sequences determined in this study and deposited in GenBank were indicated in bold; ^b^V7V8 SSU rRNA; CH Chupanga, *GO* Gorongosa.

### Detection and culture of bat trypanosomes

Bat blood samples were examined for the presence of trypanosomes by using the microhaematocrit (MH), Giemsa-stained blood smear examination and haemoculture methods as employed before for trypanosomes from different vertebrate hosts [8,10,22,33,34]. For the haemoculture, bat blood samples were transferred to tubes containing a medium consisting of solid phase blood agar base (BAB) with an overlay of LIT (liver infusion tryptose) medium containing 10% foetal bovine serum (FBS); the tubes were maintained at 25-28°C for 10–15 days. Positive cultures were transferred to culture flasks containing a monolayer of insect cells (Hi-5 from *Trichoplusia ni)* and epimastigotes from log-phase cultures were then transferred to TC100 medium (= Grace’s medium) containing 10% FBS, with incubation at 25°C. The utilization of insect feeder cells for the isolation in culture of trypanosomes largely improves the differentiation of blood trypomastigotes to epimastigotes and the multiplication of epimastigotes in primary cultures [10,33,34]. The isolates were grown in TC100 with 5.0% FBS for DNA preparation and cryopreservation at the Trypanosomatid Culture Collection (TCC) of the Department of Parasitology, University of São Paulo, Brazil.

### Amplification, sequencing and data analysis of SSU rDNA and gGAPDH

DNA was extracted from cultured bat trypanosomes by classical phenol-chloroform method and used as templates for the PCR amplification of DNA sequences. PCR amplification, cloning and sequencing of the variable V7-V8 region of SSU rRNA (employed as barcodes), whole SSU rRNA and gGAPDH genes were determined as before [35-37]. Sequences were aligned using Clustal X [38] and the resulting alignments were manually refined. We created the following alignments for phylogenetic inferences: a) the V7V8 region of SSU rRNA sequences (~880 bp) from the new bat trypanosomes aligned with their closest Australian and bat trypanosomes, yielding a high similarity index for the new bat trypanosomes by BLAST search; b) gGAPDH sequences (~830 bp) of trypanosomes representing all major clades in the phylogenetic tree of *Trypanosoma*, using non-trypanosome trypanosomatids as an outgroup; c) concatenated gGAPDH and SSU rRNA sequences (~3.3 kb) from 6 new isolates and several species of *T. cruzi* clade using *T. lewisi* as an outgroup. The species included in the phylogenetic trees and their respective host, geographical origin and GenBank accession numbers are shown in Table 1. Phylogenies were inferred by using maximum likelihood (ML), Bayesian inferences (BI) and parsimony (P) analyses. Parsimony and bootstrap analyses were carried out using PAUP version 4.0b10 [39] with 500 replicates of a random addition sequence followed by branch swapping (RAS-TBR) as previously described [36,37]. The ML analyses were performed using RAxML v.2.2.3 [40]. Tree searches were performed with GTRGAMMA, with 500 maximum parsimony starting trees. Model parameters were estimated in RAxML for the duration of the tree search. Nodal support was estimated with 500 bootstrap replicates in RAxML using GTRGAMMA and maximum parsimony starting trees. MrBayes v3.1.2 [41] was used for BI inferences as described previously [36,37].

### FFLB - Fluorescent fragment length barcoding

DNA from cultured trypanosomes and from bat blood samples were tested by FFLB carried out using four primer sets and PCR conditions described previously [10,42,43].

### Amplification, sequencing and data analysis of spliced leader (SL) sequences

The amplification and sequencing of whole SL gene repeats from bat trypanosomes were performed using primers and reaction conditions as previously described [17,27]. PCR-amplified whole SL repeats were purified from agarose gels and cloned and at least 3 clones from each isolate were sequenced. The resulting sequences were aligned with ClustalX and the resulting alignment was manually refined. The phylogenetic analysis of SL sequences was performed using the NJ method as previously described [17,19,26].

### Culture behaviour and infectivity of *T. livingstonei* for mice and triatomine insects

Two new isolates from bats (TCC1270 and 1271) were compared for their growth behaviour in TC100 and LIT media during the logarithmic and stationary phases. Cultures containing a large number of trypomastigotes at stationary phase were transferred to monolayers of HeLa cells to verify their ability to invade and develop within cells [10,22]. Epimastigotes of logarithmic cultures were transferred to monolayers of mammalian cells (LLC-MK2) and incubated at 37°C to assess the differentiation of epimastigotes into large and wide trypomastigotes resembling blood forms.

To analyse mouse infectivity, Balb/c mice were inoculated (i.p.) with *T. livingstonei* cultures containing trypomastigote forms (~10^6^/mouse) from TC100 cultures. Mouse blood samples were examined weekly from 3 to 20 days p.i. by MH, and at the 20^th^ day p.i. by haemoculture method (HE). To evaluate the behaviour of *T. livingstonei* in triatomines, 15 4^th^-5^th^ instar nymphs of each *Rhodnius neglectus* and *Triatoma infestans* were inoculated with stationary phase cultures containing epi- and metacyclic trypomastigotes, dissected at 10 and 30 days p.i., and the contents of their digestive tubes were examined for trypanosomes.

### Light, and transmission (TEM) and scanning (SEM) electron microscopy

For light microscopical analysis, blood smears from naturally infected bats and logarithmic and stationary phase cultures in TC100 medium were fixed with methanol and Giemsa-stained. For the analyses of the ultrastructural organization by TEM and SEM, cultures from two isolates were processed as previously described [10,36,37]. TEM was performed with a JEOL 100CX electron microscope. For the scanning electron microscopy (SEM), flagellates fixed with glutaraldehyde were adhered to poly-L-lysine-coated coverslips and processed for observation on a ZEISS DSM 940 microscope as previously detailed [10,34].

### Ethical approval

The capture and handling of bats was performed in accordance with the research project approved by the Scientific Boards of the Veterinary Faculty of the Universidade Eduardo Mondlane, Maputo, Mozambique and the Ethic Committee in Animal Experimentation from the Institute of Biomedical Center, University of São Paulo, São Paulo, Brazil.

## Results

### Trypanosomes in blood samples and haemocultures from bats

In this study, we evaluated trypanosome infection in 79 bats from Mozambique: 48 *Rhinolophus landeri* from Chupanga, and 31 *Hipposideros caffer* from Gorongosa (Table 1; Figure 1). We determined the Cyt b gene sequences from bat liver DNA to confirm the morphological identification and ascertain the bat species by BLAST analyses from GenBank (Table 1).

The examination of blood samples from 37 *R. landeri* by microhaematocrit revealed the presence of trypanosomes in 15 bats, yielding a prevalence of ~40%. However, the parasitemia was low and few trypomastigotes could be found in blood smears. Other blood samples from this species and from *H. caffer* could not be examined by this method because of fieldwork complications. The blood samples from all bats were examined by haemoculture, and cultures of 11 *R. landeri* isolates and three from *H. caffer* were established. These new trypanosomes from bats were first cultivated with a monolayer of Hi-5 cells in TC100 medium, and then gradually adapted to TC100 dispersing feeder cells.

### Barcoding of the new African bat trypanosomes through V7V8 rRNA sequences

**Figure 2:**
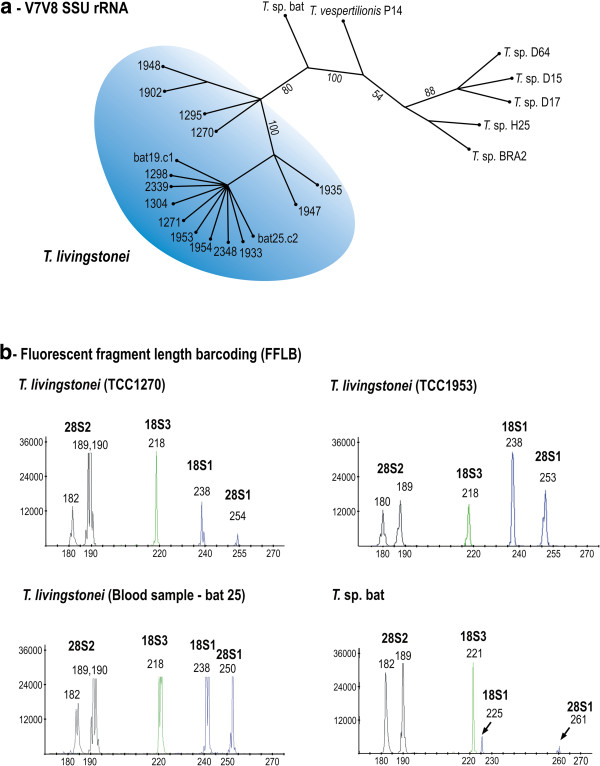
**Barcoding of new African bat trypanosomes from cultures and bat blood samples. (a)** Dendrogram inferred by using V7-V8 SSU rRNA sequences. The node numbers are bootstrap values derived from 100 replicates. **(b)** Fluorescent fragment length barcoding (FFLB) profiles of distinct isolates of the new species *T. livingstonei* and the FFLB pattern of *T.* sp. bat, a closely related bat trypanosome.

The analysis of the V7V8 variable region of the SSU rRNA gene for barcoding trypanosomes has demonstrated that this sequence is sufficiently polymorphic to distinguish all species from the several vertebrate classes examined to date [8,10,19,33,34]. In this study, barcoding using V7V8 SSU rRNA revealed that all new isolates from African bats shared high sequence similarity; 2–3 cloned sequences were determined for each isolate, and they tightly clustered together and were virtually identical (~0.2% of divergence) and different from any previously reported trypanosome species. Regarding their closest relatives, the new trypanosomes diverged ~9.5% from Australian trypanosomes from kangaroo, possums (marsupials) and rodents, and ~12% from *T.* sp. bat (Gabon, Africa) and *T. vespertilionis* (UK bat). DNA from bat blood samples with negative haemoculture results were also used as a template for barcoding, and revealed trypanosome sequences identical to those of cultivated trypanosomes (Figure 2a).

### Fluorescent Fragment Length Barcoding (FFLB) of trypanosomes from culture and blood samples

The FFLB techniques relies on the amplification and fluorescence detection of four small regions of rRNA genes of variable length according to the species/isolates, and have been valuable to distinguish a wide range of trypanosomes from cultures, blood and insect samples [10,42,43]. Here, we barcoded the new bat trypanosomes from culture and directly from blood samples. A comparison was made of their FFLB profiles with those from several previously barcoded trypanosomes, including the following species found in bats: *T. cruzi, T. c. marinkellei, T. dionisii, T. rangeli* and *T. erneyi*[10,43], resulting in unique profiles for each species. Highly similar but non-identical FFLB profiles were found for all the new cultivated bat trypanosomes and the isolates from bat blood samples. The FFLB patterns permitted to distinguish the new trypanosomes from all trypanosomes from bats and other hosts investigated in this (Figure 2b) and in previous studies [10,42,43].

### Phylogenetic analysis of new African bat trypanosomes based on gGAPDH and SSU rRNA genes

**Figure 3:**
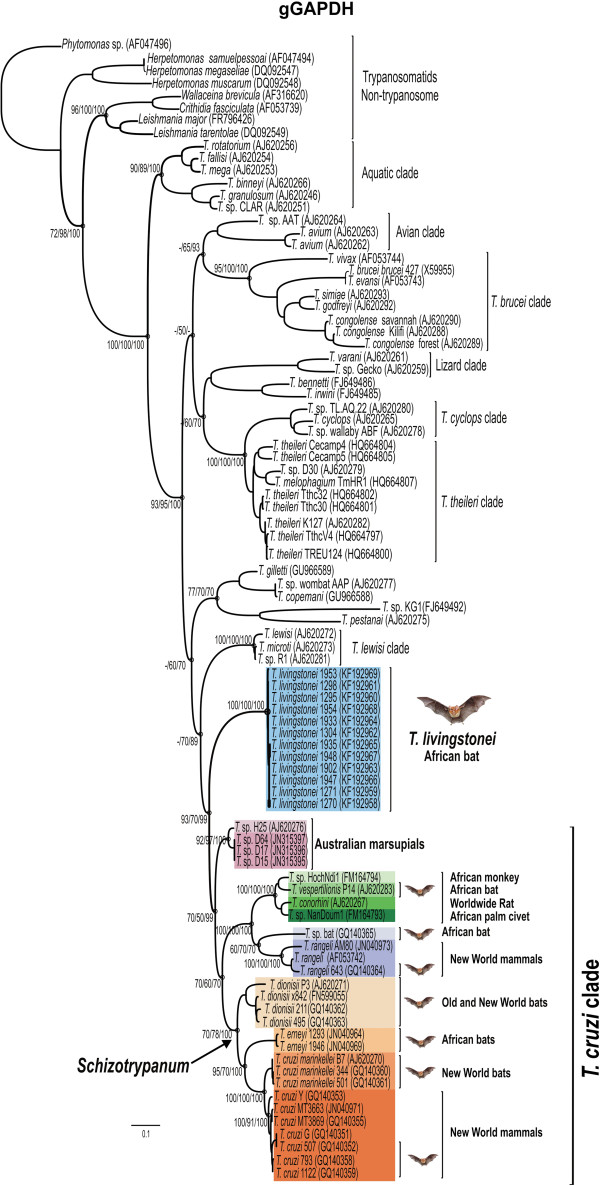
**Phylogenetic tree of the new trypanosome isolates from African bats.** Phylogenetic tree inferred by maximum likelihood (ML) of gGAPDH sequences from 12 isolates of *T. livingstonei* and 76 isolates of other species representative of all major clades with *Trypanosoma* using trypanosomatids of other genera as outgroups (832 characters, –Ln = −13552.439577). Numbers at nodes are support values derived from 500 replicates in P/ML/Bayes analyses. Codes within parenthesis are GenBank accession numbers.

Phylogenies based on SSU rRNA and gGAPDH have been used for evolutionary and taxonomic studies of trypanosomatids and it has been recommended that all new trypanosome species are phylogenetically validated using at least these two genes [10,34,36,37]. Here, the new bat isolates were initially positioned using independent gGAPDH (Figure 3) and SSU rRNA (data not shown) sequences in phylogenetic trees comprising representative species of all major trypanosome clades. Concordant tree topologies from ML, P and BI analyses were obtained by using these two genes. In all phylogenetic trees, the new bat isolates formed a well-supported clade close to Australian trypanosomes (10% divergence) and basal to the *T. cruzi* clade (Figure 3).

We selected 6 new isolates (TCC1270, 1271, 1295, 1298, 1304 and 1953) to be positioned in the phylogeny of *Trypanosoma* using concatenated data set from whole SSU rRNA and gGAPDH sequences (Figure 1). The use of these combined genes corroborated all the clades and their phylogenetic relationships as demonstrated in broader phylogenies [10,23]. The new bat isolates are highly homogeneous, diverging by only 0.3% in their gGAPDH sequences. The clade formed by the new bat trypanosomes was basal to the *T. cruzi* clade (100% bootstrap); their closest relatives were the Australian trypanosomes, whereas *T. vespertilionis* and *T.* sp. bat were more closely related, despite being separated from the new bat trypanosomes by ~13% gGAPDH sequence divergence (Figures 1 and 3).

Taken together, barcoding and phylogenetic analyses demonstrated that the new African bat isolates belong to only one species, which are exclusive to African bats so far and display intra-specific variability (genotypes) insufficient to represent more than one species. The results supported the classification of this trypanosome as a new species designated as *Trypanosoma livingstonei* n. sp., which did not belong to any known subgenus.

### Uniqueness of the primary and secondary structure of the SL gene from *T. livingstonei*

**Figure 4:**
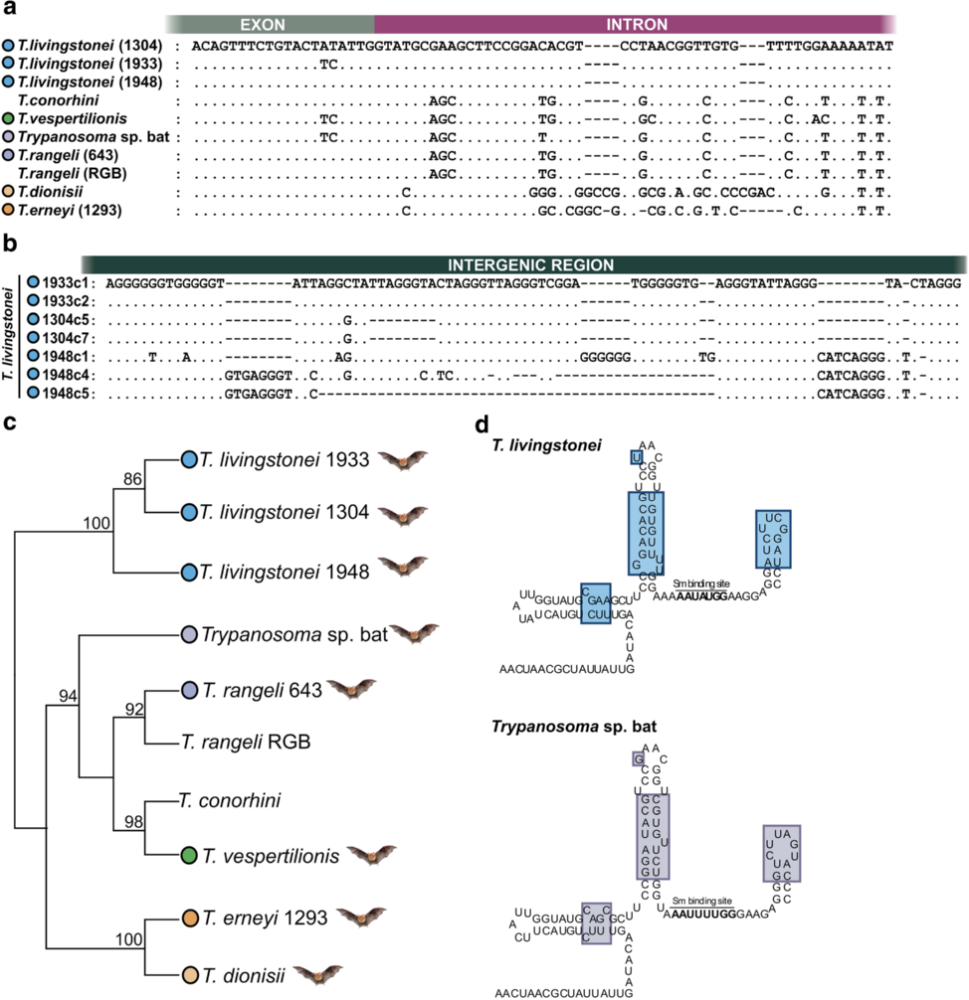
**Characterisation and phylogenetic analysis of SL genes from *T. livingstonei.*** Analysis of whole SL gene repeat (exon+intron+intergenic spacer) from isolates TCC 1304, 1933 and 1948. Comparison of primary (**a** – exon and partial intron sequences, **b** – a selected region of intergenic sequences) and secondary **(d)** structures, and relationships with phylogenetically related trypanosomes inferred using SL transcript sequences **(c)**.

We determined 3–4 sequences of full-length SL unit repeats from each of three selected isolates from *T. livingstonei.* The results showed large repeats, varying among and within the isolates as follows: 1315 bp for TCC1304, 1322 and 1326 bp for TCC1933, and 1323, 1347 and 1363 bp for TCC1948. The SL sequence alignment revealed that the 39 bp exon, which is conserved in all trypanosome species, can display different nucleotides as observed in one sequence of the isolate TCC1933 (Figure 4a), whereas the intron sequences (110 bp) were identical for all three isolates (Figure 4a). The intergenic regions were quite variable in length and sequence inter- and intra-isolates; TCC1948 showed the most highly divergent sequences (Figure 4b).

Alignments restricted to the exon and intron sequences (SL transcript) enabled an evaluation of the genetic relatedness of *T. livingstonei* with all available trypanosomes permitting reliable alignments, namely *T.* sp. bat, *T. vespertilionis, T. rangeli* and *T. conorhini. T. dionisii* and *T. erneyi* sequences could be partially aligned whereas all other species, including *T. cruzi*, resulted in inconsistent alignments. *T. livingstonei* largely diverged in their intergenic sequences from all these species (data not shown). All SL transcript sequences from *T. livingstonei* isolates clustered together and their relationships with the other species (Figure 4c) corroborated SSU rRNA and gGAPDH data (Figures 1 and 3). Additionally, we inferred the putative SL secondary structure (SL transcripts) from *T. livingstonei* and compared it with that from *T.* sp. bat. The results showed a similar general secondary structure as a consequence of their similar SL transcript sequences (Figure 4d). The SL secondary structure of *T. livingstonei* slightly differed from those inferred for *T. vespertilionis* and *T. rangeli*, whereas *T. dionisii, T. cruzi* and *T. erneyi* exhibited clearly different SL secondary structures [44]; data not show].

The sequence of whole SL repeats from *T. livingstonei* revealed a copy of the 5S ribosomal RNA (5S rRNA) gene inserted into the intergenic region in the same orientation as the SL gene. The same arrangement was demonstrated in this study on the SL repeats from *T.* sp. bat and *T. vespertilionis*. The 5S rRNA sequences from *T. livingstonei* were almost identical to those from most trypanosomes [19]. However, one nucleotide substitution (A/G at position 18) was found in the sequences from two (TCC1304 and 1948) of the three isolates from this species; this polymorphism was not detected in any other trypanosome 5S rRNA.

### Light microscopy of *T. livingstonei* blood and culture forms and behaviour in cultures

**Figure 5:**
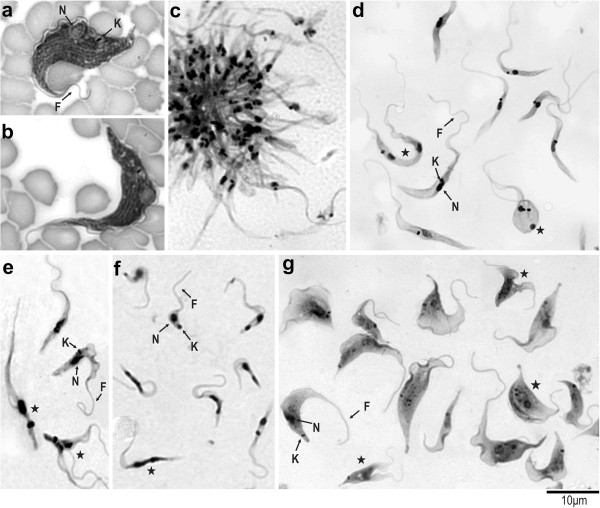
**Light microscopy of *T. livingstonei *(Giemsa-staining). (a, b)** blood trypomastigotes in naturally infected bats; epimastigotes in log-phase cultures, arranged in rosettes attached by flagella **(c)** and free epimastigotes **(d)**; large epimastigotes and trypomastigotes in mid-log cultures **(e)**; metacyclic trypomastigotes in stationary cultures **(f)**; trypomastigote forms developed in the supernatant of LLC-MK2 mammalian cell monolayers at 37°C **(g)**. Dividing flagellates are indicated by stars. Nucleus (N); kinetoplast (K) and flagellum (F). Scale bars: 10 μm.

Trypanosomes found in bat blood from which the *T. livingstonei* isolates were derived were large trypomastigotes with a broad body and a pointed posterior end, a markedly frilled undulating membrane and a short free flagellum. The small kinetoplast occupied a marginal position adjacent to the rounded and nearly central nucleus and several surface striations (Figure 5a,b). Dividing forms were not observed in blood smears.

The first forms observed in haemoculture were epimastigotes arranged in rosettes and attached by their flagella (Figure 5c). Free epimastigotes in the supernatant predominated during the log- and mid-phase cultures, and these forms are pleomorphic, with bodies varying in length from 16.0 to 29.0 μm (with an average of 22.3 μm) and from 1.1 to 2.0 μm in width (average of 1.56 μm), with most forms displaying long free flagellum (average 15.5 μm length) (Figure 5d). Both rounded forms and large epimastigotes were observed as dividing forms at log-phase (Figure 5d). Flagellates from the mid-log cultures are mostly large epimastigotes with a prominent undulant membrane and long flagella, and they were long and slender trypomastigotes; both are dividing forms (Figure 5e). At stationary cultures, small and slim flagellates with a large and nearly terminal kinetoplast resembling metacyclic trypomastigote forms were predominant (Figure 5f).

When co-cultivated with a monolayer of HeLa mammalian cells at 37°C, *T. livingstonei* epimastigotes developed into wide free-swimming trypomastigotes in the supernatant (Figure 5g), with a resemblance but a smaller size than the bat blood forms (Figure 5a,b). These trypomastigotes were dividing in early cultures (Figure 5g), but attempts to successively culture them were unsuccessful. The development of epimastigotes into broad trypomastigotes under similar culture conditions is a feature of *Megatrypanum* spp. [1,45]. *T. livingstonei* was unable to invade and develop within mammalian cells, a feature of all *Schizotrypanum* species [10].

### Scanning and transmission electron microscopy of *T. livingstonei*

**Figure 6:**
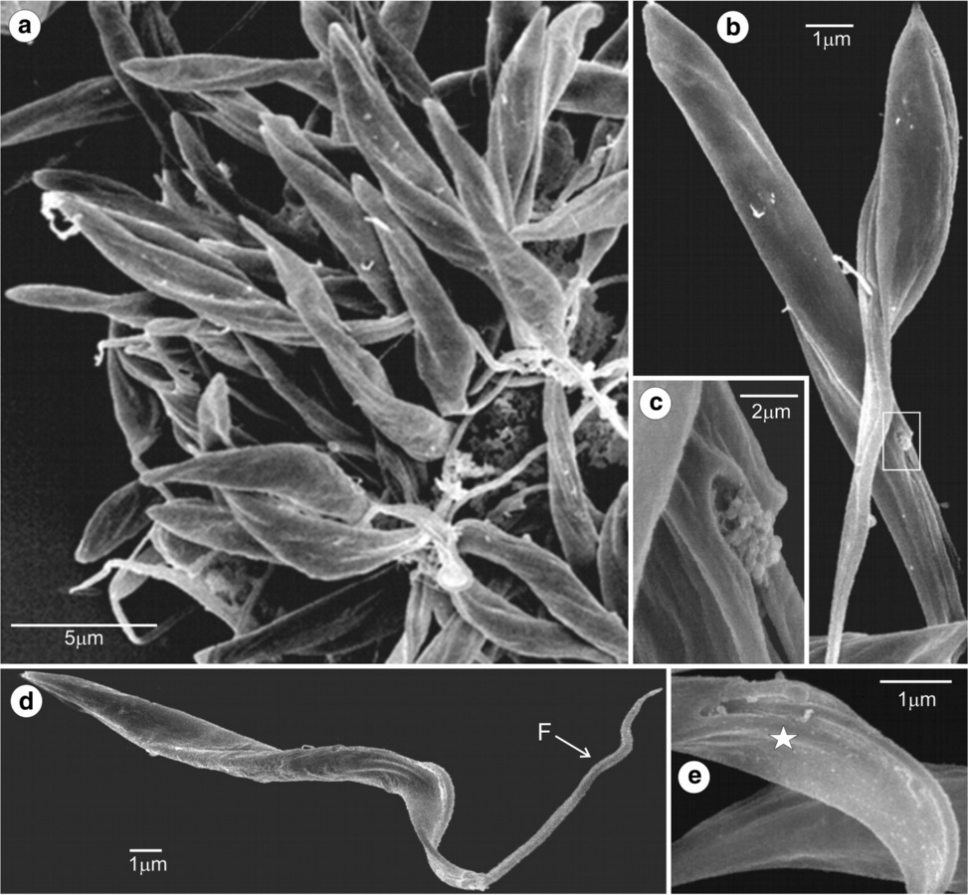
**Scanning electron microscopy of *T. livingstonei*. (a)** Epimastigotes attached by their flagella forming a rosette; **(b)** free-swimming epimastigote flagellar pocket and the emergence of the flagellum; **(c)** detail of the ruffled region with the cytostome opening; **(d)** long, slender and twisted epimastigote; **(e)** dilated anterior extremity formed by joined cell and flagellar membranes is indicated by stars.

Scanning electron microscopy (SEM) analyses of *T. livingstonei* cultures showed a diversity of forms including the following: a) large rosettes of epimastigotes united by flagella with one to three body torsions (Figure 6a); b) a ruffled area near the cytostome, an invagination of the membrane close to the flagellar pocket shown by SEM as a small opening near the emergence of the flagellum (Figure 6b,c); c) long and slender epimastigotes with noticeable body torsions and a dilated anterior extremity constituted by the joining of the flagellum and cell membranes (“undulant membrane”) before the emergence of the flagellum (Figure 6d,e).

**Figure 7:**
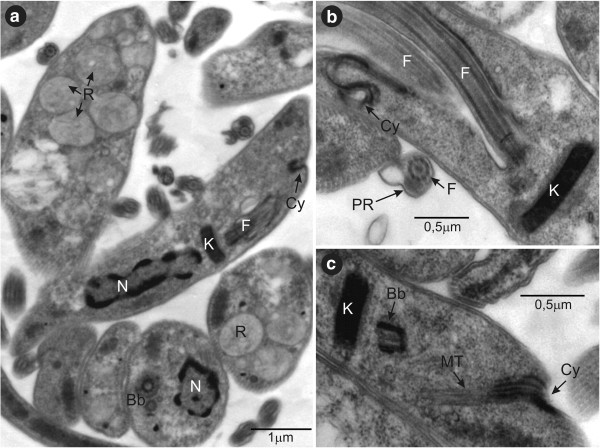
**Transmission electron microscopy (TEM) of *T. livingstonei*. (a)** Ultrastructural organisation of epimastigotes in longitudinal and transversal sections showing the nucleus, kinetoplast, reservosomes concentrated at the posterior region, basal body, flagellum and paraflagellar rod **(b)** longitudinal section of an dividing epimastigote exhibiting a new emerging flagella and a lengthened kinetoplast and the invagination of the flagellar pocket membrane forming the cytostome; **(c)** longitudinal section showing the cytostome opening and groove surrounded by microtubules penetrating deep into the cytoplasm; **(c)** compacted disk shaped kinetoplast. N, nucleus; K, kinetoplast; F, flagellum; R, reservosome; Cy, cytostome; Bb, basal body; PR, paraflagellar rod; MT, microtubules.

The ultrastructural organisation of *T. livingstonei* epimastigotes (TEM analysis) revealed all common organelles of trypanosomatids. However, some features should be mentioned as follows: a) The cytostome (Figure 7a,b,c), which forms together with the flagellar pocket the main structure involved in the endocytic process; b) a large number of reservosomes, which are compartments that accumulate endocytosed macromolecules found at the posterior region of epimastigotes (Figure 7a); c) the compacted disk-shaped kinetoplast structure (Figure 7a,b,c).

### *T. livingstonei* behaviour in mice and triatomines

The small trypomastigotes from end-phase *T. livingstonei* cultures (TC100 medium)*,* which most likely correspond to metacyclic forms, were incapable of infecting Balb/c mice. This absence of infection was confirmed by blood examination using the microhaematocrit from two to 15 days post-inoculation and, after that, by haemoculture. Epi- and trypomastigotes were unable to infect triatomines (*Rhodnius neglectus* and *Triatoma infestans*).

### Taxonomic summary

**New species description**: Phylum Euglenozoa, Cavalier-Smith, 1981; class Kinetoplastea, Honigberg, 1963; order Trypanosomatida (Kent, 1880) Hollande, 1952; family Trypanosomatidae, Doflein, 1951. *Trypanosoma livingstonei* Teixeira and Camargo n. sp.

**Type material**: hapantotype: culture TCC1270. Paratypes: cultures TCC1271, 1295, 1298, 1304, 1902, 1933, 1935, 1947, 1948, 1953, 1954, 2339 and 2348, whose bat hosts and locality of collection in Mozambique, Africa, are in Table 1. **Type host**: Chiroptera, Rhinolophidae, *Rhinolophus landeri*. **Additional host**: Chiroptera, Hipposideridae, *Hipposideros caffer.***Locality**: Mozambique, Province of Sofala, District of Chupanga (S18°02′ E35°34′), Zambezi valley and the Gorongosa National Park (S18°58′ E34°21′). **Morphology**: *T. livingstonei* exhibits large and wide blood trypomastigotes (average 32.4 μm length and 7.8 μm wide) with several striations, small kinetoplast, frilled undulating membrane and a free flagellum averaging 11.0 μm in length. Epimastigotes from log-phase cultures are mostly slender and pointed at posterior ends, ranging from 16.0 to 29.0 μm length and 1.1 to 2.0 μm wide, with free flagellum averaging 15.5 μm length; the kinetoplast in general is close to the nucleus. **Diagnosis**: DNA sequences unique to *T. livingstonei* are deposited in GenBank (accession numbers): SSU rRNA (KF192979 - KF192994), gGAPDH (KF192958 - KF192969) and SL gene (KF192970 - KF192976). Cultures are cryopreserved at the Trypanosomatid Culture Collection of the University of São Paulo, TCC-USP. Glass slides of Giemsa-stained smears from bat blood samples and cultures and DNA samples are also kept at TCC-USP. To comply with the regulations of the International Code of Zoological Nomenclature (ICZN), details of this species have been submitted to ZooBank with the Life Science Identifier (LSID) zoobank.org:pub: D8714C8C-71F5-44ED-AC91-2106E49C1A6D.

**Etymology***:* The name was given because *Trypanosoma livingstonei* n. sp. was first discovered in bats captured in Chupanga, Mozambique, a small village in the margin of the Zambezi River, where Mary Livingstone, the wife of David Livingstone, died of “fevers” in 1862; her grave remains in an small cemetery from a Portuguese Mission practically destroyed by the Mozambique wars.

## Discussion

For a better appraisal of the genetic diversity and evolutionary history of trypanosomes, and for their reliable classification and phylogenetic inferences, studies must include trypanosomes from all vertebrate classes, representative of orders, genera and species, by using molecular phylogenetic approaches. Bats are among the most common hosts of a large variety of trypanosomes in Africa, Asia, South America and Europe. However, our knowledge of their genetic diversity, hosts, vectors, life cycles, pathology, distribution and phylogenetic relationships is restricted to a few species. Almost all available data are about the species of the subgenus *Schizotrypanum* because bat trypanosomes in this subgenus are the closest relatives of the human pathogen *T. cruzi*[8-11,13,14,22,25,28,46]. However, several bats around the world harbour a plethora of trypanosome species, most of which are morphologically assigned to the subgenus *Megatrypanum*[1,2,4,6,7,47].

In this study, we surveyed trypanosomes in blood samples from bats of old world-restricted families Rhinolophidae and Hipposideridae captured in Mozambique. We obtained 11 haemocultures from *R. landeri* and 3 from *H. caffer*. Morphologically, the large trypomastigotes found in bat blood smears would be assigned to the subgenus *Megatrypanum.* However, multilocus phylogeny validated in this subgenus only the trypanosomes from ruminants allied to *T. theileri*[16,17,19,45]. With the exception of artiodactyls, bats were the main hosts of trypanosomes morphologically classified in the subgenus *Megatrypanum*[1,7], so a thorough phylogenetic analysis of bat trypanosomes was required to warrant their exclusion from this subgenus.

The phylogenetic positioning of *T. livingstonei* and *T.* sp. bat, both of which are morphologically compatible with the subgenus *Megatrypanum*, support the exclusion of bats as hosts of species in this subgenus. The morphology of *T. livingstonei* blood and culture forms largely differs from those of the *Megatrypanum* species. However, epimastigotes of this species developed into large trypomastigotes resembling blood forms under a monolayer of mammalian cells at 37°C, a process also observed for the *Megatrypanum* trypanosomes [1,45]. In fact, *T. livingstonei* blood and culture forms exhibited unique morphological features as shown by light and SEM microscopy. This new species exhibited a cytostome, reservosomes and a disk-shaped kinetoplast, all of which are absent in species of the *Megatrypanum* and common to those of *Schizotrypanum*. This is the first study to use TEM and SEM to analyse a bat trypanosome not classified into *Schizotrypanum*. There are no unambiguous differences in the overall ultrastructural organisation that would be useful for distinguishing *T. livingstonei* from *Schizotrypanum* trypanosomes, even though the new species strongly diverged with all molecular markers and in several biological features such as the inability to develop inside mammalian cells and lack of infection in mice and triatomine bugs.

The vectors of *T. livingstonei* are so far unknown; in this work we demonstrated its inability to infect *T. infestans* and *Rhodnius neglectus* in accordance with the fact that triatomines cannot be the vectors of this species because they do not occur in Africa. In Africa and Europe, bat bugs (cimicids) are the vectors of *T*. *dionisii* and *T*. (*Megatrypanum) incertum*[6,7]. The bat restricted *Stricticimex brevispinosus* was found to be infected by a *Megatrypanum* trypanosome in Africa [48]. In addition, sand flies were incriminated as vectors of *T. (Megatrypanum) leonidasdeanei* in South America [49].

The barcoding of new African bat trypanosomes morphologically assignable to the subgenus *Megatrypanum* through both V7V8 SSU rRNA and FFLB has shown similar sequences and profiles for all the new isolates, which were shown to be highly different from the barcodes of other trypanosomes from bats and other hosts [10,42,43]. In all the inferred phylogenetic analyses, the new bat trypanosomes always tightly clustered together, forming a homogeneous clade separated by sufficient genetic distances from all other trypanosomes to allow their description as a new species, that is, *T. livingstonei* n. sp.*,* which does not nest within any known subgenera*.*

For insect and plant trypanosomatids, SL genes have proven to be valuable for identifying the genera and species of cultivated flagellates, as well as for the barcoding of trypanosomatids directly from their hosts [50-53]. SL RNA genes have also been used for species identification and genotyping of *T. cruzi* and *T. rangeli*[22,26,27,54], *T. vivax*[55] and *T. theileri*[17,19]. The characterisation of whole SL gene repeats in *T. livingstonei* showed a larger length and more polymorphic sequences among isolates of the same species and repeats of the same isolate, when compared to other trypanosome species. In addition, this species enclosed a copy of 5S rRNA within its intergenic region, as reported before for *T. vivax*, *T. conorhini, T. rangeli*, *T. desterrensis, T. theileri* and *T. melophagium*, but absent in *T. cruzi, T. cruzi marinkellei, T. brucei* and *T. lewisi*[17,19,21,44,45,55,56]. Here, the comparison of primary and secondary structures from the SL rRNA of *T. livingstonei* and other trypanosomes corroborated its close relationships with the trypanosomes that nested into a strongly supported (despite being highly heterogeneous) major clade containing African, Europe and South American species from bats (*T.* sp. bat and *T. vespertilionis), T. rangeli, T. conorhini* and trypanosomes from monkeys and civets.

The trypomastigotes we found in blood smears from bats infected with *T. livingstonei* resembled those denominated as *T. heybergi-*type and described for the African *Megatrypanum* trypanosomes *T. leleupi, T. mpapuense, T. morinorum* and *T*. *thomasi*. These species, which could all be synomies, were reported in bats from Congo, Zambia, Kenya and Tanzania [1,3,7,47]. Our findings corroborated that African species of *Rhinolophus* and *Hipposideros* bats harbour trypanosomes morphologically similar to *T. heybergi*. However, *T. (Megatrypanum) leonidasdeanei* and *T. (Megatrypanum) pessoai* were reported in South American bats and also described as resembling *T. heybergi*[49,57]. Nevertheless, no cultures, DNA sequences or blood smears were available from any *T. heybergi*-type trypanosomes, which prevented the molecular comparison between previously reported species and our new isolates.

Bat species harbouring *T. livingstonei* are endemic to sub-Saharan African bats, although their genera, *Rhinolophus* and *Hipposideros,* are widespread throughout Asia, Oceania, Europe and Africa, but both are absent from the New World*.* Bats may have originated in Laurasia (~ 65 MYA), and bat trypanosomes should have diverged since the great diversification/expansion of bats in the Eocene [29-31]. A long past and extensive bat radiation, recent movement of bats across large geographic distances (even large oceanic barriers but not across the Atlantic Ocean), and incomplete bat palaeontology have complicated the studies about the origin and dispersion of bat trypanosomes. Associations between bats and their trypanosomes, and an evaluation of possible paleontological and eco-biogeographical scenarios could account for the origin, genetic diversity, relationships and current distribution of these parasites and are crucial for understanding their evolutionary history.

There is an urgent need for an extensive taxonomic revision of the genera *Trypanosoma* on a strongly supported phylogenetic basis that firstly requires the molecular analyses of a large sampling representative of host species and geographic ranges. This may allow for the description of several new species and the creation of new subgenera to accommodate new species that formed clades without correspondence to any subgenera previously proposed by Hoare [1]. To meet these objectives, new trypanosome cultures should be obtained and deposited in reference collections. The naming of any new trypanosomatid species should be considered valid only when supported by sound and broad phylogenies (using at least SSU rRNA and gGAPDH genes). However, the description of new trypanosome species based on small DNA sequences, accompanied or not by the morphology of blood flagellates (mostly because cultivation have failed), have been accepted [58-60]. We are designating the new African bat isolates as *T. livingstonei* on the basis of its position in the *Trypanosoma* phylogenetic trees inferred using SSU rRNA and gGAPDH genes, its genetic distances from other species and also taking into account its peculiar SL RNA gene repeats. Morphological features and information regarding host species, and its behaviour in culture and in mice complement the species description. These data can be valuable for comparative studies of the cellular biology, host-parasite interactions, ecology and evolution of trypanosomes.

## Conclusion

The phylogenetic evidence produced by this study underscores the great genetic diversity of trypanosomes in bats around the world. *T. livingstonei* fell at the edge of the *T. cruzi* clade, which comprises all bat trypanosomes sampled to date regardless of whether they are from Africa, Europe or South America. The position of *T. livingstonei* at the base of the *T. cruzi* clade further supports the hypothesis that the clade was ancestrally a group of bat-restricted parasites that evolved exclusively in these hosts and later jumped at independent times to mammals of other orders. In the most likely scenario, the trypanosomes from several mammalian orders nested into this clade, including those from African and Australian terrestrial mammals*,* evolved from a bat trypanosome. Other explanations require multiple jumps into bats, which seem less probably. Apparently, this ancestral bat trypanosome gave rise morphologically, biologically (different life cycles and vectors), ecologically and genetically distinct species. The positioning of *T. livingstonei* in all inferred phylogenies provides evidence that the *T. cruzi* clade derived from a bat trypanosome, prior to the splits between *T. cruzi*, *T. rangeli* and the Australian groups, hence, lends further support to the bat seeding evolutionary hypothesis for the origin of this clade [24]. This study also adds some additional support to *T. cruzi* itself evolving from a bat trypanosome as the resulting data make more likely that the common ancestor of *T. rangeli* and *T. cruzi* was a bat trypanosome.

## Competing interests

The authors declare that they have no competing interests.

## Authors’ contributions

LL, EPC and MMGT conceived the study and designed the experiments; LL, OEA, PBH, LN, CSAT, MC, MA, WS assisted with sample collection, performed the experiments and analyzed the data; LL, PBH, EPC, MMGT prepared the paper. All authors read, revised and approved the submitted version of the manuscript.
